# Supplementary material for: Modeling glioblastoma heterogeneity as a dynamic network of cell states
Source: Mol Syst Biol. 2021 Sep 16;17(9):e10105. doi: 10.15252/msb.202010105 (PMC8444284; doi:10.15252/msb.202010105)
Supplement: Supplementary file 5 — Source Data for Figure 3 [file MSB-17-e10105-s001.zip › Figure3A_sourcedata/GSEA_3065/nosigdb_state1.GseaPreranked.1622718409535/RICHARDS_NEFTEL_CELL_2019_AC.html]

Details for gene set RICHARDS\_NEFTEL\_CELL\_2019\_AC[GSEA]

|  || Dataset | state1 |
| Phenotype | NoPhenotypeAvailable |
| Upregulated in class | na\_neg |
| GeneSet | RICHARDS\_NEFTEL\_CELL\_2019\_AC |
| Enrichment Score (ES) | -0.8178701 |
| Normalized Enrichment Score (NES) | -2.3323393 |
| Nominal p-value | 0.0 |
| FDR q-value | 0.0 |
| FWER p-Value | 0.0 |
Table: GSEA Results Summary

  

Fig 1: Enrichment plot: RICHARDS\_NEFTEL\_CELL\_2019\_AC      
 Profile of the Running ES Score & Positions of GeneSet Members on the Rank Ordered List

  

| PROBE | GENE SYMBOL | GENE\_TITLE | RANK IN GENE LIST | RANK METRIC SCORE | RUNNING ES | CORE ENRICHMENT || 1 | S100A16 |  |  | 27 | 0.587 | 0.0368 | No |
| 2 | DBI |  |  | 110 | 0.352 | 0.0522 | No |
| 3 | METTL7B |  |  | 355 | 0.239 | 0.0435 | No |
| 4 | ANXA5 |  |  | 709 | 0.171 | 0.0191 | No |
| 5 | RAB31 |  |  | 7256 | -0.057 | -0.6418 | No |
| 6 | RAMP1 |  |  | 7655 | -0.068 | -0.6776 | No |
| 7 | HOPX |  |  | 7850 | -0.075 | -0.6923 | No |
| 8 | MT3 |  |  | 8954 | -0.139 | -0.7949 | No |
| 9 | GATM |  |  | 9049 | -0.147 | -0.7945 | No |
| 10 | HEY1 |  |  | 9215 | -0.168 | -0.8000 | No |
| 11 | PMP22 |  |  | 9392 | -0.199 | -0.8045 | Yes |
| 12 | ATP1B2 |  |  | 9441 | -0.210 | -0.7952 | Yes |
| 13 | CST3 |  |  | 9581 | -0.254 | -0.7922 | Yes |
| 14 | PCDHGC3 |  |  | 9588 | -0.255 | -0.7757 | Yes |
| 15 | PLTP |  |  | 9727 | -0.336 | -0.7671 | Yes |
| 16 | MLC1 |  |  | 9728 | -0.338 | -0.7443 | Yes |
| 17 | SPARC |  |  | 9738 | -0.347 | -0.7219 | Yes |
| 18 | HEPN1 |  |  | 9782 | -0.395 | -0.6997 | Yes |
| 19 | CLU |  |  | 9795 | -0.418 | -0.6727 | Yes |
| 20 | SPARCL1 |  |  | 9798 | -0.423 | -0.6444 | Yes |
| 21 | S100B |  |  | 9822 | -0.507 | -0.6126 | Yes |
| 22 | SLC1A3 |  |  | 9823 | -0.509 | -0.5783 | Yes |
| 23 | EDNRB |  |  | 9824 | -0.509 | -0.5441 | Yes |
| 24 | FABP7 |  |  | 9825 | -0.513 | -0.5095 | Yes |
| 25 | PON2 |  |  | 9835 | -0.554 | -0.4732 | Yes |
| 26 | PRCP |  |  | 9855 | -0.712 | -0.4272 | Yes |
| 27 | PTPRZ1 |  |  | 9869 | -0.918 | -0.3667 | Yes |
| 28 | PMP2 |  |  | 9871 | -0.982 | -0.3007 | Yes |
| 29 | TTYH1 |  |  | 9872 | -1.033 | -0.2311 | Yes |
| 30 | GPM6B |  |  | 9873 | -1.091 | -0.1577 | Yes |
| 31 | GFAP |  |  | 9877 | -1.174 | -0.0790 | Yes |
| 32 | BCAN |  |  | 9878 | -1.175 | 0.0001 | Yes |
Table: GSEA details [plain text format]

  

Fig 2: RICHARDS\_NEFTEL\_CELL\_2019\_AC: Random ES distribution      
 Gene set null distribution of ES for **RICHARDS\_NEFTEL\_CELL\_2019\_AC**
